# Groups and Emotional Arousal Mediate Neural Synchrony and Perceived Ritual Efficacy

**DOI:** 10.3389/fpsyg.2018.02071

**Published:** 2018-10-26

**Authors:** Philip S. Cho, Nicolas Escoffier, Yinan Mao, April Ching, Christopher Green, Jonathan Jong, Harvey Whitehouse

**Affiliations:** ^1^Yonsei University, Underwood International College, Songdo, South Korea; ^2^Yonsei University, Institute of Convergence Science, Center for Science and Engineering Applications in Social Science, Seoul, South Korea; ^3^Department of Psychology, National University of Singapore, Singapore, Singapore; ^4^Life Sciences Institute Programme in Neurobiology and Aging, National University of Singapore, Singapore, Singapore; ^5^Department of Statistics, National University of Singapore, Singapore, Singapore; ^6^MARCS Institute, Western Sydney University, Penrith, NSW, Australia; ^7^Wayne State School of Medicine, Diagnostic Radiology and Psychiatry, Detroit, MI, United States; ^8^Center for Psychology, Behavior and Achievement, Coventry University, Coventry, United Kingdom; ^9^Institute of Cognitive and Evolutionary Anthropology, University of Oxford, Oxford, United Kingdom

**Keywords:** brain, interpersonal synchrony, religion, ritual, efficacy

## Abstract

We present the first neurophysiological signatures showing distinctive effects of group social context and emotional arousal on cultural perceptions, such as the efficacy of religious rituals. Using a novel protocol, EEG data were simultaneously recorded from ethnic Chinese religious believers in group and individual settings as they rated the perceived efficacy of low, medium, and high arousal spirit-medium rituals presented as video clips. Neural oscillatory patterns were then analyzed for these perceptual judgements, categorized as low, medium, and high efficacy. The results revealed distinct neural signatures and behavioral patterns between the experimental conditions. Arousal levels predicted ratings of ritual efficacy. Increased efficacy was marked by suppressed alpha and beta power, regardless of group or individual setting. In groups, efficacy ratings converged. Individual setting showed increased within-participant phase synchronization in alpha and beta bands, while group setting enhanced between-participant theta phase synchronization. This reflected group participants' orientation toward a common perspective and social coordination. These findings suggest that co-presence in groups leads to a social-tuning effect supported by between-participant theta phase synchrony. Together these neural synchrony patterns reveal how collective rituals have both individual and communal dimensions. The emotionality of spirit-medium rituals drives individual perceptions of efficacy, while co-presence in groups signals the significance of an event and socially tunes enhanced agreement in perceptual ratings. In other words, mass gatherings may foster social cohesion without necessarily requiring group-size scaling limitations of direct face-to-face interaction. This could have implications for the scaling computability of synchrony in large groups as well as for humanistic studies in areas such as symbolic interactionism.

## Introduction

A central question in the history of the human sciences has been how social groups and emotions shape perceptions of shared culture, such as religious rituals. Anthropologists and sociologists have long argued that the communality and emotionality of a rituals bind social groups in the collective belief in a performance's meaningfulness and efficacy (Durkheim, 1995; Collins, 2004; Whitehouse, 2004; Scheve and Salmela, 2014; Di Paolo and De Jaegher, 2015). But, efforts to disentangle the social, psychological, and especially neurophysiological mechanisms involved have been limited in volume and scope.

Social scientists have long debated over the role of emotions in the perception of ritual efficacy. In his pioneering fieldwork on the Trobriand Islands, Bronislaw Malinowski argued that ritual efficacy derived from a desire to control situations where outcomes are uncertain, as a means of reducing anxiety (Malinowski, 1935, 1945; Felson and Gmelch, 1979). Subsequent studies have shown the emotional motivations of rituals such as praying, chanting, and using lucky charms in the face of acute failure or annihilation (Case et al., 2004; Burger and Lynn, 2005; Rudski and Edwards, 2007). Building on the insights of Levi-Strauss, medical sociologists and psychiatrists have argued that emotions evoked by rituals, within the multifaceted symbolism of myths, elicit a sense of meaningfulness for participants (Dow, 1986; Kirmayer, 2004; Lee, 2010).

The emotional arousal associated with many collective rituals has been investigated as a primary factor in forging tight communal bonds and identity fusion (Whitehouse, 2004; Swann et al., 2012; Jackson et al., 2018). In one study of a highly intense firewalking ritual, participants' increase in heart rates correlated with the rate of audience members who were socially close to them (Konvalinka et al., 2011). Such raw emotional response was important for the common individual experience of the ritual among group members. Arousal may further enhance the perception of rituals for religious believers who have greater cultural competence and sensitivity to particular symbolic cues (McCauley and Lawson, 2002). Together, this suggests that emotional arousal is important for the perception of rituals, especially in their social aspects.

Interestingly, emotional arousal has been found to synchronize cortical networks for visual attention and emotional processing across individually tested participants (Nummenmaa et al., 2012). Hasson et al. found that individual brains “tick collectively” producing “intersubject synchronization,” when presented with the same stimuli of arousing movie scenes (Hasson et al., 2004, 2008). These intersubject correlations extended into high-order association areas and may facilitate interpersonal understanding and synchronization in groups. While these studies suggest that social and neural synchronization linked to arousal may play an important role in the perception of rituals, this possibility has not yet been investigated.

Another important factor in group rituals is co-presence. Co-presence in groups has been shown by social psychologists to produce a social-tuning effect. Evidence comes from studies where participants in a group social setting, or who simply believed an event was being shared by others, agreed more in their recalled judgements (Higgins, 1978; Gigerenzer, 2004; Higgins et al., 2007; Shteynberg, 2010; Shteynberg et al., 2014). In other words, this effect emerged based on what individual participants assumed was common experience with similar others. Such social tuning, even without communication, is likely highly relevant to ritual perception and may be supported by common neural states across participants (Echterhoff et al., 2009; Shteynberg, 2010).

While co-presence has been shown to enhance agreement in emotional perceptions (Golland et al., 2015), little work has examined whether co-presence alone without explicit interaction triggers a social-tuning effect and neural synchronization. Co-presence may increase the perceived likelihood of social engagement and anticipated joint-attention, resulting in activation of cognitive processes related to social coordination and perception. Recent studies of joint-action and joint-attention have shown that such a common neural state emerges as two-person synchronization during coordinated physical movement or attentional gaze (Sebanz et al., 2006; Grossmann et al., 2007; Sanger et al., 2011; Metcalfe and Terrace, 2013). For example, social coordination during a range of interactions from musical performance to hand-waving has resulted in inter-participant theta-phase synchronization (Lindenberger et al., 2009; Yun et al., 2012). No studies however have addressed the fundamental question of whether group as opposed to individual experience of emotionally intense collective rituals synchronizes neural states.

Our primary objective was to address the outstanding questions discussed above by examining behavioral and neurological signatures for religious believers' perception of ritual efficacy. The effects of co-presence and emotional arousal during rituals that are associated with these signatures need closer examination. Both behavioral and neurophysiological measures are needed to elucidate the interaction of emotions and social setting in collective rituals. We analyze behavioral ratings and associated neural signatures in brain wave oscillatory power, as well as within-participant and between-participant phase synchrony.

A complication with some earlier studies on arousal and co-presence, such as Konvalinka et al. (2011) and Golland et al. (2015), is that all participants were only tested in group setting, and without an adequate group or individual control. Group participants might show synchrony merely because they experience the same stimuli, leading to the similar and synchronized stimulus-related brain response across all participants. This synchronization does not appear because of co-presence, but simply because stimulus onset is the same for all members of the group. The same stimulus-related synchronization would appear when participants are alone and experiencing the same stimuli. To solve this issue, we introduce a control condition where participants experience the ritual alone, and compare this to the effect of co-presence in a group condition, while controlling for the identical stimulus input.

To examine arousal and social tuning in groups we studied an ancient form of religious rituals still widely practiced, Chinese spirit-mediumship. This type of ritual was ideal to investigate these questions for two reasons. First, the most salient and ubiquitous characteristic of most Chinese spirit-medium rituals is their highly arousing, bloody, and emotionally intense performances, often involving self-mutilation (Chan, 2006; Boretz, 2011; Cho, 2013). Second, they involved both collective and individual aspects, with audiences both collectively viewing the ritual's theatrical performance and individually consulting the medium.

In our experiment, EEG oscillations and behavioral ratings of perceived ritual efficacy were recorded from ethnic Chinese religious believers viewing short video clips of Chinese spirit-medium rituals. Social setting was manipulated by having participants seated individually or in a group of three. As discussed above, we were careful to control for the potential issues in previous studies and wanted to separate stimulus-related synchrony from group-related synchrony. To evaluate stimulus related synchrony we created virtual triads from individually tested participants, with the synchrony measured in these virtual triads only due to the stimulus-related synchrony. If groups showed enhanced synchrony as compared to individuals in virtual triads, this meant the synchrony observed in groups occurred on top of stimulus-related synchrony and really only emerges in groups.

To examine ritual arousal's effect on perceptions of efficacy, religious believers rated the perceived efficacy of ritual performances in videos categorized as low, medium, and high arousal, based on independent ratings by non-believers. This was important because we wanted to investigate how arousal influences religious judgments, and we had to obtain normative arousal ratings that were independent of any religious judgement. In addition to efficacy, religious believers also rated two additional cultural constructs of the ritual performance, the perceived degree of the spirit-medium's possession and intentional agency.

To examine the neural signature correlates of the perception of rituals, the video clips were categorized and analyzed according to the experimental participants' judgements of low, medium, and high perceived efficacy. EEG power was analyzed within participant and EEG phase synchrony both within and between participants using the phase locking value (PLV) (Lachaux et al., 1999).

We first hypothesized that the degree of ritual arousal should predict religious believers' perceptual ratings of ritual efficacy. This is because some believers report that more intense ritual acts reflect to them the spirit-medium's potency as he is supposedly more deeply connected with the god. Perceptions of ritual efficacy should thus be positively correlated with ratings of the spirit-medium's degree of possession and negatively correlated with ratings of the spirit-medium's intentional agency. In the neural signatures, we expected this to result in decreased power in both alpha and low beta bands, as markers of increased arousal and attention (Wrobel, 2000; Aftanas et al., 2001; Sarlo et al., 2005; Sauseng et al., 2005).

Second, we hypothesized that co-presence in group social setting would signal the cultural significance of an event and would both alter attention and produce a social-tuning effect. In other words, the presence of others, which frames the meaningfulness of the ritual event, would alter how attention is deployed toward them, beyond the effect of the arousing aspects of the rituals (Hanslmayr et al., 2009). In addition, social tuning should result in greater agreement among participants' perceptual ratings of ritual efficacy in group as compared to individual setting.

Moreover, we expected that co-presence in groups would also produce similar neural patterns and synchrony among participants. Based on the synchrony literature, the common pattern of responses to rituals, even without explicit social interaction, may be reflected by increased between-participant phase synchrony in groups. Theta band is a primary candidate for this effect because it has been linked to social coordination (Cavanagh et al., 2009; Min and Park, 2010; Staudigl and Hanslmayr, 2013).

## Materials and methods

### Participants

Thirty-six Chinese adults (18 females, 18 males, mean age = 44.1, SD = 14.9) participated in the study. They were recruited through advertisement circulated among followers of Chinese temples located in Singapore and in which spirit-mediums perform. Participants were included based on self-reported prior exposure to and belief in Chinese spirit-medium rituals. Procedures were approved by the Institutional Review Board of the National University of Singapore and all methods performed according to relevant guidelines and regulations. Participants signed informed consent prior to the study and were financially compensated for their time.

### Stimuli

Video material was taken from recordings of Chinese spirit-mediums performing different rituals, such as entering trance, spirit-writing, and forms of self-mutilation (e.g., tongue-cutting) (Supplementary Videos 1–3). From each recording, 5 s clips were cut out. They covered all phases of each ritual (i.e., beginning, middle, and end), and therefore showcased actions of variable emotional content. A total of 177 clips were collected and converted to a resolution of 640 × 320 pixels (24 frame per seconds, MPEG-4 AVC compression at 2.3 Kbits/s).

The emotional responses to the clips were then evaluated by 20 ethnic Chinese raters (9 females, 11 males, mean age = 23.8, SD = 2.4) who did not participate in the main experiment. They reported no exposure to, or belief in, Chinese spirit-medium rituals. This allowed us to assess the emotional content of the video independently of knowledge of mediumistic rituals. Participants rated their emotional response to the videos on dimensions of valence and arousal using a 10-point Self-Assessment Manikin scale (SAM, Bradley and Lang, 1994). Based on Arousal ratings we selected 105 videos split in low arousal, medium arousal, and high arousal categories (35 videos each; resp. arousal ratings: 2.4, SD = 4.2; 3.9, SD = 2.4; 5.4, SD = 4.0). Categories were built so there were significant between-categories difference in arousal ratings (all pairwise *ps* < 0.001), and no significant between-categories differences in average luminance (*ps* > 0.99) and root-mean square contrast (*ps* > 0.52).

### EEG and behavioral experiment methods

#### Procedure

The experiment was conducted in two different social settings: a group setting, and an individual setting. In the group social setting, 3 participants were seated together at the left, right, and center of a single screen. In the individual social setting, participants were tested individually, and seating position alternated across participants among the left, right, and center positions. Viewing distance was identical for all participants (1.1 m). The participants seated on the left and right viewed the screen at a 45° angle. Viewing position and gender were counterbalanced across conditions and participants. After participants were seated, they were given instructions about their task. It consisted of watching the video clips, and after each, making five judgments using 10 points scales. Participants were asked to rate Efficacy (how efficacious, éİĹ *ling*, was the ritual), the degree of spirit-medium's possession (how possessed was the medium), the degree of spirit-medium's intentional agency (how much the spirit-medium intended to do what he was doing) and their emotional response to the video on dimensions of Arousal and Valence using the SAM (Bradley and Lang, 1994). They gave their responses using a keyboard placed on their lap. Response hand was identical for all participant of a group and it was counterbalanced across groups; in individual settings it was counterbalanced across participants. Participants were asked to look at the screen at all times.

The main experiment session started with a task practice on three videos. This was followed by 105 experimental trials. During each trial a fixation cross (2 s) was presented followed by video presentation on a black background (5 s duration, apparent size = 18.4° × 9.1°). A cue for response including rating description and visual scale was shown for each of the 5 ratings. It stayed on screen until after all participants had responded. Arousal and Valence were always presented first after each video, followed in random order by the Efficacy, Possession and Agency ratings. The trial ended when all participants had given the last rating. Inter-trial interval consisted in a blank screen of duration varying randomly between 0.5 and 1 s. There were two 30 s breaks, one after each third of the trials. The main experiment session lasted about 50 min on average.

At the end of the experimental session each participant was seated separately and filled in a questionnaire recording basic demographic information and assessing their degree of exposure to Chinese spirit-medium rituals. Participants were then compensated for their time, and debriefed.

#### Group and individual matching

An important concern was that differences in seating position, video presentation order, response hand, and sex ratio between individual and group participants could confound the effect of social setting. To avoid this possibility we equated these variables between individual and group setting. This was done so that individual participants could ultimately be grouped into virtual triads, that matched one-to-one a Group triad with respect to these variables. Consequently a given seating position, presentation order, and response hand appeared equally frequently in both settings. Participant sex was counterbalanced across triads for groups and matching virtual triads for individuals: 2 triads were all male, 2 triads were all female, 1 triads had one male and two females, and 1 triads had 2 males and 1 female.

### Data processing and analysis

#### Behavior

To investigate whether the emotional content of a ritual as rated by non-believers could predict ratings by believers, we analyzed religious believers' perceptual ratings of efficacy using a mixed ANOVA with within-subject factors Arousal (low, medium, high) and between-subject factor Social setting (group, individual).

Two-tailed tests were used for all follow-up tests unless noted otherwise. We used one-tailed *t*-tests for testing comparisons between individual and groups. We made this choice because, unlike other effects, social setting was tested between participants and we wanted to overcome the loss of statistical power that this entailed. This was further motivated by the fact that we were careful to recruit participants from devout spirit-medium communities, rather than just the general ethnic Chinese population, to ensure the authenticity of their belief and common cultural background. True believers tended to be elderly and unwilling to participate in an experiment because some felt that doing so might offend their god. This made obtaining a larger sample size extremely challenging. We considered that a possible trade-off on type 1 error was compounded by the fact that we had directional hypothesis derived from the literature for all of these tests, and that they were only performed when warranted by a significant ANOVA effect. We used Welch tests when appropriate. Standardized effect sizes are reported as generalized eta squared for ANOVAs, and Cohen's d_z_ for *t*-tests. Multiple comparisons were dealt with by controlling the false discovery rate (FDR) at 5% over all tests that were run in the study. Unless otherwise noted tests had a FDR q-value lower than or equal to 0.05, and therefore passed a 5% FDR criterion (Storey, 2002).

#### EEG data recording and preprocessing

**Figure 1:**
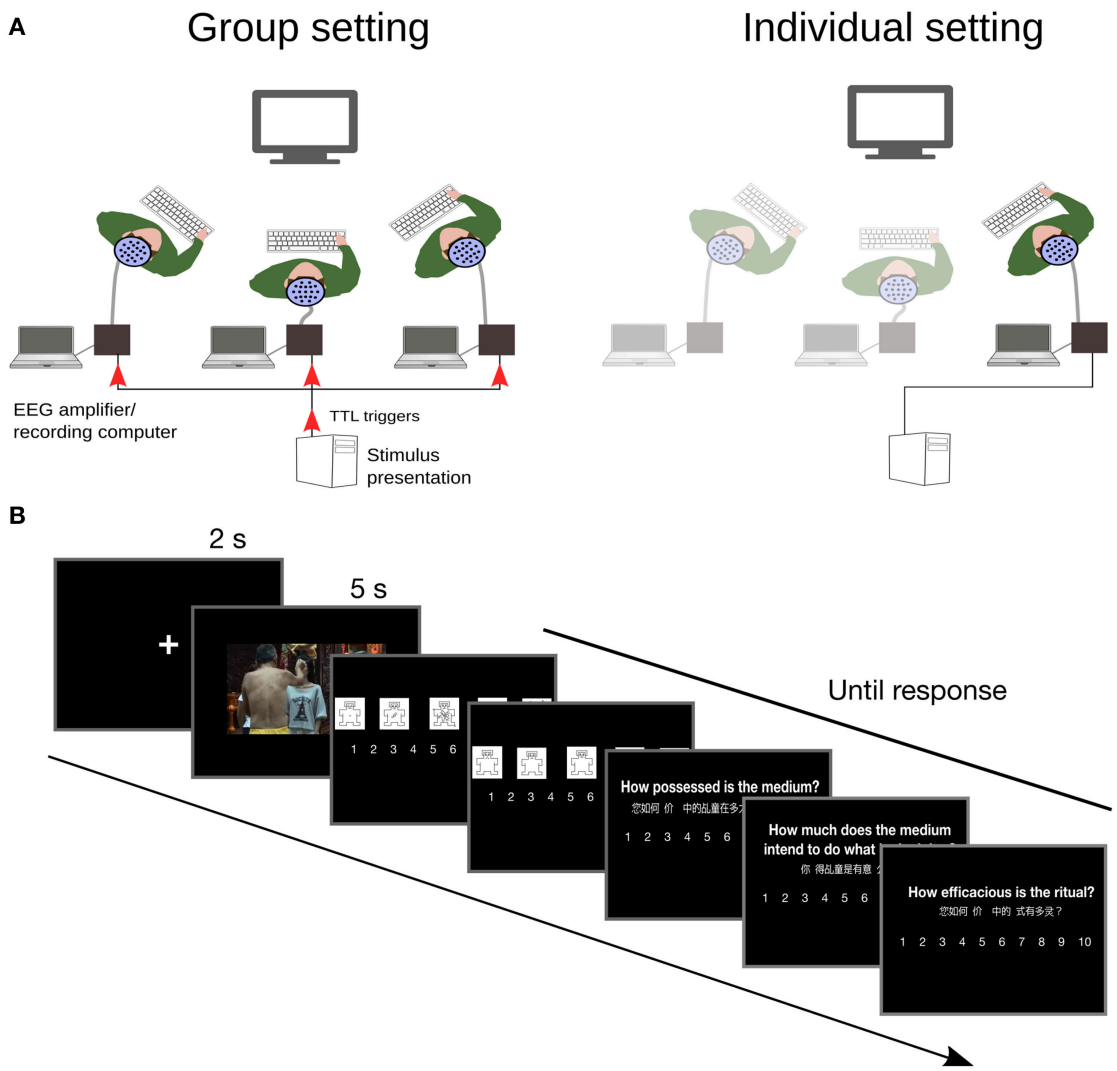
Experimental setup and timeline. **(A)** Religious believers rated the perceived efficacy of Chinese spirit medium rituals presented in video clips, while electroencephalographic data were recorded. In the group social setting, all participants attended to the same screen. In the individual social setting, participants watched video clips alone. **(B)** Experimental timeline.

Electroencephalographic data were recorded from 19 Ag/AgCl scalp electrodes mounted in an elastic cap according to the modified 10/20 system. Electrodes covered left and right hemispheres at frontal (Fp1, F3, F7; Fp2, F4, F8), central-lateral (C3, T7; C4, T8), and posterior regions (P3, P7, O1; P4, P8, O2), and midline (Fz, Cz, Pz). EEG signal was recorded at 256 Hz referenced to the nose. Vertical and horizontal EOGs were assessed using recordings at Fp1 and Fp2, and F7 and F8, respectively (Amenedo and Escera, 2000). Data in individual setting were recorded using a 24-channels amplifier (ANT neuro). In the group social setting, EEG data were simultaneously recorded from three participants using two 24-channels and one 72-channels amplifier (ANT neuro). Synchronization of recordings between devices was achieved by sending parallel port triggers at the onset of each trial to all three amplifiers simultaneously (see Figure 1).

The EEG signal was preprocessed with EEGLAB (Delorme and Makeig, 2004). It was filtered off-line using low-pass (stop band edge at 0.2 Hz) and high-pass (stop band edge at 100 Hz) sinc filters, and re-referenced to a 19 channels average reference. Data were epoched time-locked to video onset, with a 1.5 s pre-stimulus interval and a 5.5 s post-stimulus interval.

Epoched data were visually examined, and epochs with excessive movement and muscle activity artifacts were rejected. Artifacts related to eye blinks, eye movements, and cardiac activity were then removed using independent component analysis (ICA, Jung et al., 2000). After ICA decomposition, independent components that captured artifactual activity were visually identified and pruned. The data were then back-projected before further analysis.

#### EEG wavelet decomposition

EEG epochs were subjected to a continuous Morlet wavelet transform (Tallon-Baudry et al., 1997) as implemented in EEGLAB. We used a set of wavelets that captured frequencies from 4 Hz to 80 Hz (c = 3–24, increasing linearly; σf = 2.6–6.7 Hz; σt = 136–942 ms). This covered our frequency bands of interest theta (4–7), alpha (8–12), low beta (13–20), high beta (21–30), low gamma (31–50), and high gamma (51–80).

#### EEG power and phase synchrony analysis

Raw power was computed from the wavelet transform at each frequency and time point. Individual trial values were then averaged, scaled by average baseline power and transformed to a decibel scale [10^*^log(signal)]. The baseline interval was chosen between 750 ms and 250 ms before stimulus onset to prevent overlap with stimulus onset activity (Herrmann et al., 2014).

Phase synchrony was investigated to assess the degree of temporal alignment of brain activity between regions of interest. This was performed both within and across participants. For each frequency band of interest phase values were derived from wavelet parameters. The degree of phase synchrony between channels was assessed across trials using the phase locking factor (PLV, Lachaux et al., 1999). The PLV quantifies the degree of time-locking of oscillatory phase of two channels at each frequency and time point. It varies between 0 and 1, with higher value reflecting increased phase synchrony between channels. The PLV at time (t) was computed as

(1) $$\begin{array}{r} {\text{PLV}}_{\text{t}}=\frac{1}{N}|\overset{N}{\underset{n=1}{\sum }}e^{\left(i\theta \left(t,n\right)\right)}| \end{array}$$

where, θ(t, n) is the phase difference between the two channels at time t and trial n.

For within-participant synchrony analyses, within-participant PLV was calculated between 171 channels pairs from the 19 channels within each participant, excluding pairing each channel with itself. For between-participant synchrony analyses, channels pairs were taken between each pair of participants. We computed the PLV between all possible 190 pairings between the 19 channels of each participant. This was performed for all 3 possible pairs of participants in each triad, and averaged across pairs to give a summary measure of phase locking for each triad at each frequency and time point. For participants in individual settings, the same procedure was applied but with participants grouped into virtual control triads as previously described.

For between-participant analysis, we were concerned by the potential confound of 50 Hz power line noise. Because participants in group setting are recorded simultaneously, line noise, if present, would have the same phase across participants. This would not be the case in individual settings because recordings were on separate days and the phase of the noise would differ across participants. Hence, spurious differences in phase synchrony could be observed between the two groups. To investigate and control for that possibility we used several processing strategies (see Supplementary Datasheet).

#### EEG statistical analysis

The goal of the EEG analysis was to identify the neural correlates of the perceived Efficacy of spirit-medium rituals, and explore their modulation by Social setting. We aggregated trials into 3 categories of increasing perceived efficacy (low, medium, and high) constructed using a percentile split of the clips based on average efficacy ratings recorded during the experiment.

Data were averaged over 4 s post-stimulus, excluding the first second, which excluded the early transient responses evoked by stimulus onset that were not of interest here (Makeig et al., 2002; Gruber et al., 2005). In addition, this prevented the analysis to be affected by the smearing of baseline activity into the post-stimulus interval.

All oscillatory power and synchrony data were analyzed for each frequency band separately using ANOVAs with within-subject factors Efficacy (low, medium, high), and between-subject factor Social setting (individual, group).

We also investigated topographical effects using additional factors, which were constructed differently for power and synchrony analysis. For power, topographical differences were investigated using an additional Region of Interest (ROI) factor. It included the following regions: anterior left (al: Fp1, F3, F7), anterior right (ar: Fp2, F4, F8), central left (cl: C3, T7), central right (cr: C4, T8), posterior left (pl: P3, P7, O1), posterior right (pr: P4, P8, O2), and central (z: Fz, Cz, Pz). An illustration of ROI groupings is presented in Figure S1 in Supplementary Datasheet. For each of these regions, data were averaged over electrodes. These regions were selected so they would provide a distributed coverage of the scalp so that topographical effects could be assessed (Schirmer et al., 2016).

For both within- and between-participant synchrony analysis, topographical effects were investigated using an additional factor built using k-means clustering that grouped ROI pairs into clusters. Clusters represent groups of ROI pairs that are highly correlated across variables of interest and that capture functionally equivalent patterns of connectivity (Deen et al., 2011). We chose this approach because the aggregation of channel pairs into ROI pairs led to a ROI factor with 28 levels, which resulted in ANOVAs models complexity that was beyond our computational resources. Clustering was done separately for within and between synchrony data. Examinations of scree plots indicated that a choice of 4 clusters was optimal as it was parsimonious while returning homogeneous, low variance, clusters (see Supplementary Datasheet for within- and between-participant ROI-clusters of channel pairs and scree plots). Clusters are presented in **Figure 3** for illustrative purposes.

Consistent with the strategy we used for behavior analysis, follow-up tests were 2-tailed except for group differences, for which one-tailed tests were used.

## Results

### Behavioral results

Overall, the ratings of efficacy were highly correlated with the other measures of ritual perception we recorded during the experiment. They correlated positively with ratings of spirit-medium possession (*r* = 0.96, df = 103, *p* < 0.001), and negatively with ratings of spirit-medium intentional agency (*r* = −0.74, df = 103, *p* < 0.001). This indicated that the ratings were an adequate measure of participants' evaluation of the rituals.

**Figure 2:**
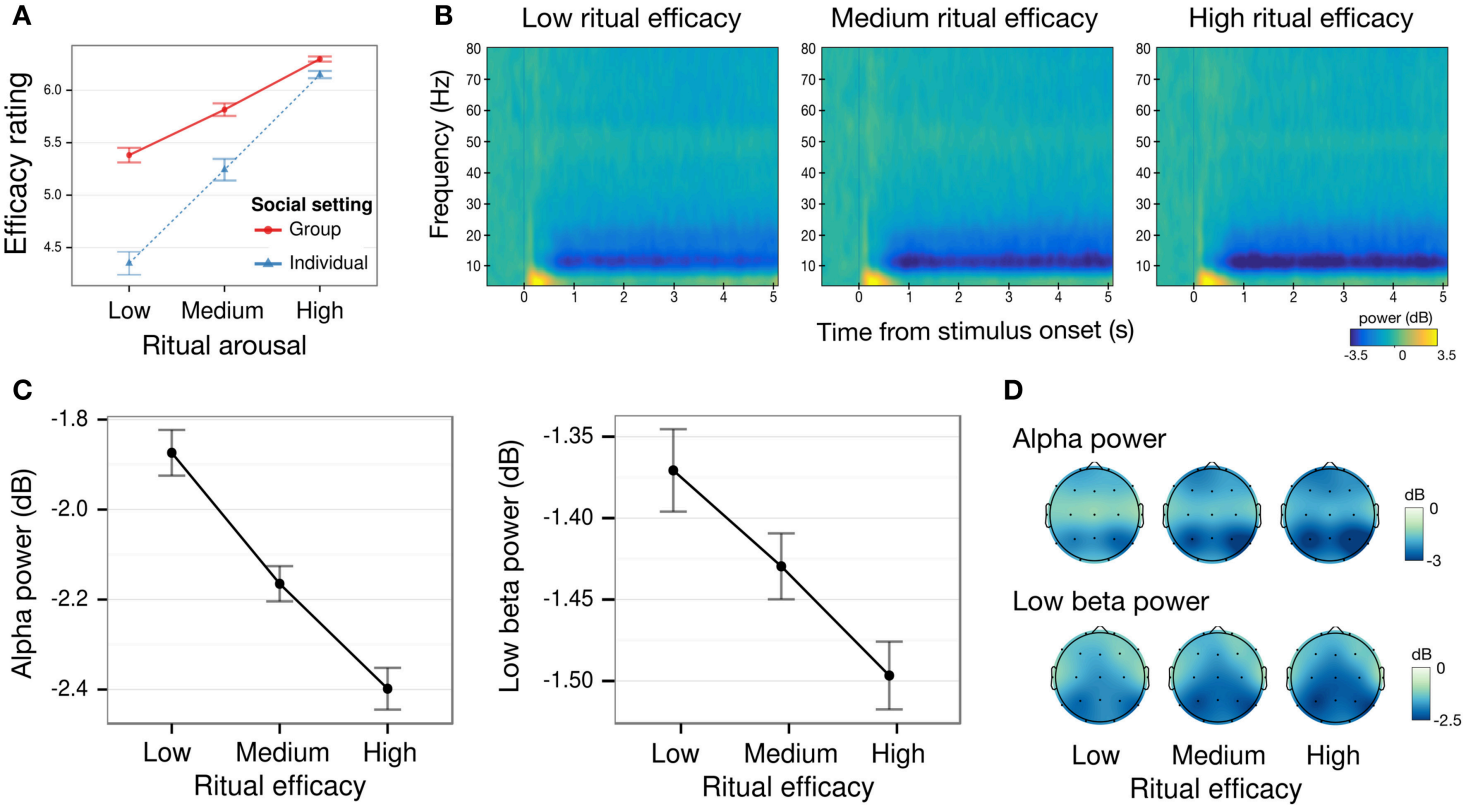
Behavioral and oscillatory power correlates of the perceived efficacy of Chinese spirit-medium rituals. **(A)** Mean behavioral ratings of perceived ritual efficacy by arousal and social setting. Emotionally arousing rituals were rated as significantly more efficacious. Efficacy ratings for low arousal rituals were marginally higher when viewed in a group of three participants than individually. **(B)** Time-frequency plot of mean power changes from baseline for low, medium, and high efficacy rituals. **(C)** Mean alpha (8 – 12 Hz) and low beta (13 – 20 Hz) power decrease from baseline over all electrodes by perceived ritual efficacy, showing decreasing power for increasing efficacy (1 – 5 s post-stimulus onset window) **(D)** Alpha and low beta power topography (1 – 5 s post-stimulus onset window). All error-bars indicate within-participant standard error of the mean. Where there was no significant difference between individual and groups settings, their data were aggregated before plotting.

We investigated whether Arousal predicted enhanced perceived Efficacy, and whether this effect interacted with Social setting (Figure 2). Repeated measures ANOVA with Arousal (low, medium, high) as a within-subject factor and Social setting (individual, group) as a between-subject factor, revealed a significant main effect of Arousal [*F*_(2, 68)_ = 42.11, *p* < 0.001 η^2^ = 0.10]. The effect of Arousal was present in both individuals and groups. Perceived efficacy was rated higher for high arousal as compared to both medium arousal videos [group: *t*_(17)_ = 3.45, *p* = 0.003, *d*_*z*_ = 0.81; individual: *t*_(17)_ = 4.5, *p* < 0.001, *d*_*z*_ = 1.05], and low arousal videos [group: *t*_(17)_ = 4.39, *p* < 0.001, *d*_*z*_ = 1.04; individual: *t*_(17)_ = 5.25, *p* < 0.001, *d*_*z*_ = 1.24]. The same pattern was observed between medium arousal videos and low arousal videos [group: *t*_(17)_ = 4.47, *p* < 0.001, *d*_*z*_ = 1.05]; individual: *t*_(17)_ = 5.5, *p* < 0.001, *d*_*z*_ = 1.19].

While there was no significant overall main effect of Social settings [*F*_(1, 34)_ = 1.56, *p* = *0*.220, η^2^ = 0.04], participants' perceptual ratings of efficacy were nonetheless affected when they were in groups, as indicated by a significant two-way interaction of Arousal and Social setting [*F*_(2, 68)_ = 4.47, *p* = *0*.015, η^2^ = 0.01]. Efficacy ratings were marginally higher for group as compared to individuals for low arousal [*t*_(34)_ = 1.64, *p* = 0.056, *d*_*s*_ = 0.55, one-tailed], but not for medium arousal [*t*_(34)_ = 1.03, *p* = 0.16, *d*_*s*_ = 0.35, one-tailed] and high arousal videos [*t*_(34)_ = 0.30, *p* = 0.38, *d*_*s*_ = 0.10, one-tailed].

To test for the hypothesized enhanced convergence in participants' rating in the group as compared to the individual setting, we ran an F-test of equality of variances of participants' judgments. Between-subject variance of Efficacy ratings was reduced in the group setting (SD = 1.49) as compared to the individual setting (SD = 1.99), decreasing by about nearly 60% [*F*_(53, 53)_ = 0.57, *p* = 0.020], indicating a convergence of ratings in groups.

### EEG power

In order to identify the neural correlates of perceived ritual Efficacy we investigated EEG power in the theta, alpha, beta, and gamma bands. We conducted mixed effects ANOVAs with the between-subject factor Social setting (individual, group), and within-subject factors Efficacy (low, medium, high) that was based on a percentile split of participants' efficacy ratings, Region of Interest (ROI: anterior right AR, anterior left AL, central right CR, central left CL, posterior right PR, posterior left PL, midline ML), and for beta power analysis only, a Band factor (high, low) that differentiated low and high beta. Power results are presented in Figure 2.

In the theta band, there was a main effect of ROI cluster [F_(6, 204)_ = 8.43, *p* = 0.003, η^2^ = 0.045], which reflected a posterior distribution for theta oscillations. The main effect of Efficacy was not significant [F_(2, 68)_ = 1.19, *p* = 0.311, η^2^ = 0.002; other effects are *ps* > 0.090].

In the alpha band (Figures 2B,C), there was a significant effect of Efficacy [*F*_(2, 68)_ = 8.11, *p* < 0.001, η^2^ = 0.01]. Alpha power decreased significantly for high efficacy [*t*_(35)_ = 3.61, *p* < *0.0*01, *d*_*z*_ = 0.60] and medium efficacy videos [*t*_(35)_ = 2.29, *p* = *0.0*28, FDR q-value = 0.066, *d*_*z*_ = 0.45] as compared to low efficacy videos, and between high efficacy and medium efficacy videos [*t*_(35)_ = 2.06, *p* = 0.047, FDR q-value = 0.102, *d*_*z*_ = 0.34]. The main effect of ROI [*F*_(6, 204)_ = 14.70, *p* < 0.001, η^2^ = 0.037] was significant, reflecting typical posterior alpha topography (other effects *ps* > 0.18)(Figure 2D).

In the low beta band (Figures 2B,C), a comparable main effect of Efficacy was observed [*F*_(2, 68)_ = 4.68, *p* = 0.013, η^2^ = 0.01], with decreased power for high efficacy [*t*_(35)_ = 2.64, *p* = *0.0*12, *d*_*z*_ = 0.44] as compared to low efficacy videos, and no significant power differences for medium efficacy videos as compared to both high efficacy [*t*_(35)_ = 1.42, *p* = 0.16, *d*_*z*_ = 0.23] and low efficacy videos [*t*_(35)_ = 1.78, *p* = 0.084, *d*_*z*_ = 0.30].

In the high beta band, the effect of Efficacy was not significant [*F*_(2, 68)_ = 0.20, *p* = *0.8*2, η^2^ = 0.001]. The effect of ROI was significant for both low [*F*_(6, 204)_ = 19.62, *p* < 0.001, η^2^ = 0.11] and high beta band [*F*_(6, 204)_ = 8.21, *p* < 0.001, η^2^ = 0.07] reflecting a posterior distribution. No other effects were significant in the beta bands (*ps* > 0.07).

In the gamma band, there were no significant effects (*ps* > 0.21) other than a main effect of ROI [*F*_(2, 68)_ = 6.08, *p* < 0.001, η^2^ = 0.04] with higher gamma power at anterior and posterior sites.

### Within-participant phase-locking value (PLV)

**Figure 3:**
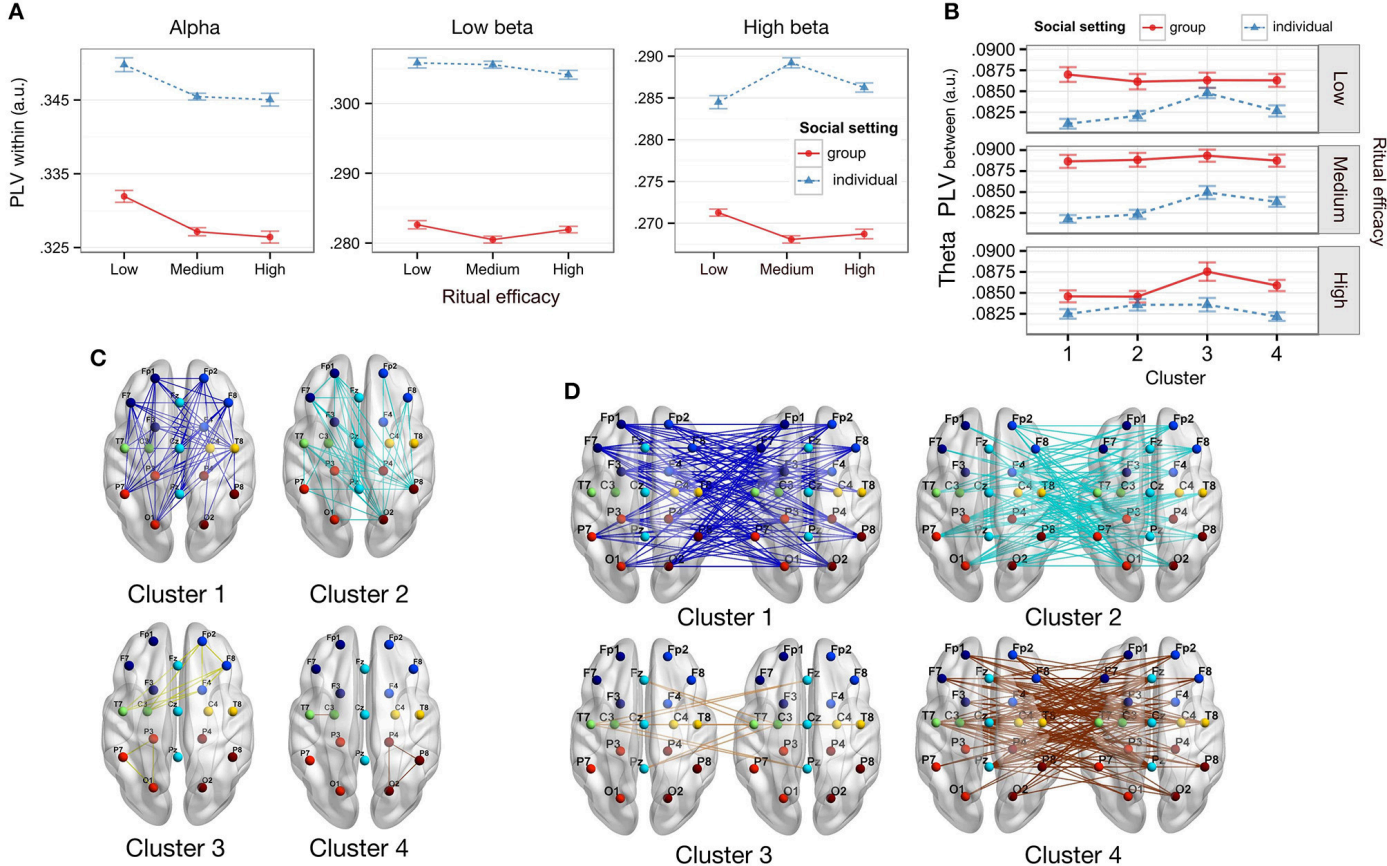
Within- and between-participant phase synchrony correlates of perceived ritual efficacy. **(A)** Within-participant PLV by efficacy and social setting. Alpha band PLV decreased for low efficacy rituals. Low beta PLV decreased in the group as compared to individual setting. High beta PLV decreased in the group Social setting for medium and high efficacy rituals. **(B)** Theta between-participant PLV by Efficacy and Social setting. Group setting heightens between-participant PLV over individual setting in theta band for low efficacy in anterior and posterior ROI (cluster 1) **(C)** Within-participant ROI PLV clusters. **(D)** Between-participant ROI PLV clusters. Error bars represent within-participant standard error of the mean.

The neural synchrony correlates of perceived ritual Efficacy were first investigated within-participants by examining within-participant PLV. For each frequency band, ANOVAs were conducted with within-participant factor Efficacy (low, medium, high), between-participant factor Social setting (individual, group), and an additional ROI cluster factor which captured differences between independent topographical clusters (4 clusters, see methods section for details on cluster construction) see Figures 3A,C.

In theta band, there was a main effect of Efficacy [*F*_(2, 68)_ = 6.397, *p* = 0.003, η^2^ = 0.002]. PLV was significantly higher for low Efficacy compared to medium and high Efficacy [*t*_(35)_ = 2.42, *p* = 0.021, *d*_*z*_ = 0.40; *t*_(35)_ = 3.36, *p* = 0.002, *d*_*z*_ = 0.56], but not between medium and high Efficacy (*p* > 0.355). There was also an effect of Cluster [*F*_(3, 102)_ = 68.39, *p* < 0.001, η^2^ = 0.573], with lowest PLV observed in Cluster 1 and the highest in Cluster 4. Intermediate PLV values were observed in cluster 2 and 3, which were not significantly different from each other (*p* = 0.092). All other pairwise cluster differences were significant (*p* < 0.001).

In the alpha band (Figure 3A), there was a significant main effect of Efficacy [*F*_(2, 68)_ = 6.899, *p* = 0.002, η^2^ = 0.001]; follow-ups indicated that PLV was significantly higher for low efficacy videos as compared to both medium efficacy [*t*_(35)_ = 3.47, *p* = 0.001, *d*_*z*_ = 0.58] and high efficacy videos [*t*_(35)_ = 2.75, *p* = 0.009, *d*_*z*_ = 0.46]. There was no alpha PLV difference between medium and high efficacy videos [*t*_(35)_ = 0.47, *p* = 0.64, *d*_*z*_ = 0.078]. Other effects were non-significant (*ps* > 0.18) other than a significant ROI cluster effect [*F*_(3, 105)_ = 60.55, *p* < 0.001, η^2^ = 0.499]. Follow-up tests showed that PLV in cluster 4 (central left to central left; and posterior right to posterior right) was significantly higher than any other cluster (*ps* < 0.001).

In the low beta band (Figure 3A), there was a main effect of Social setting [*F*_(1, 34)_ = 4.57, *p* = 0.040, FDR q-value = 0.090, η^2^ = 0.037], but no effect of Efficacy [*F*_(2, 70)_ = 0.73, *p* = 0.49, η^2^ = 0.02]. PLV was significantly higher in the individual compared to the group Social setting [*t*_(35)_ = 2.14, *p* = 0.020, *d*_*z*_ = 0.73, one-tailed] (other effects *ps* > 0.45).

In high beta (Figure 3A) there was a significant two-way interaction of Efficacy by Social setting [*F*_(2, 68)_ = 6.00, *p* = 0.004, η^2^ = 0.001]. Follow-up tests showed that PLV for participants in the individual social setting was significantly higher compared to participants in the group social setting, for medium efficacy [*t*_(35)_ = 2.16, *p* = 0.019, *d*_*z*_ = 0.73, one-tailed] and high efficacy [*t*_(35)_ = 1.86, *p* = 0.036, FDR q-value = 0.082, *d*_*z*_ = 0.63, one-tailed], but not for low efficacy rituals [t_(35)_ = −1.39, *p* = 0.087, *d*_*z*_ = −0.47, other effects *ps* > 0.077, one-tailed].

In the high gamma band there was a three-way interaction of Social setting, Efficacy, and ROI-cluster [*F*_(6, 204)_ = 3.65, *p* = 0.002, η^2^ = 0.001]. However, when clusters were analyzed independently, there were no significant interaction of Social-setting and Efficacy (other effects *ps* > 0.19).

In low gamma band there was no significant effect (*ps* > 0.369).

### Between-participant phase locking value

We investigated how efficacy altered patterns of neural synchrony *between* participants, by examining the between-participant PLV. For each frequency band, ANOVAs were carried out with between-participant factor Social setting (individual, group) and within-participant factors Efficacy (low, medium, high) and ROI cluster (4 levels) see Figures 3B,D.

There were significant effects in the theta band only (other bands *p* > 0.086). Results were consistent across three different processing strategies (see methods), and we present the findings for data processed using a notch filter.

There was a significant effect of Cluster [*F*_(3, 102)_ = 11.77; *p* < 0.001, η^2^ = 0.006] linked to higher PLV in cluster 3 compared to other clusters [*t*s_(35)_ > 3.28, *ps* < 0.002, *d*_*z*_> 0.426] and in cluster 4 as compared to cluster 1 [t_(35)_ = 2.56, *p* = 0.015, *d*_*z*_ = 0.462].

Importantly this effect was qualified by a three-way interaction of Social setting, Cluster, and Efficacy [*F*_(6, 204)_ = 3.43; *p* = 0.003, η^2^ = 0.004]. A breakdown of the three-way interaction by Efficacy revealed a significant interaction between Social setting and Cluster for low efficacy only [*F*_(3, 104)_ = 6.55; *p* < 0.001, η^2^ = 0.005], with PLV enhanced in the group over individual Social setting in cluster 1 [t_(35)_ = 1.72, *p* = 0.047, FDR q-value = 0.102, *d*_*z*_ = 0.357, one-tailed], and no other clusters [*ps* > 0.13]. The interaction was not significant for other conditions [*ps* > 0.08].

Interestingly, cluster 1 activity was in anterior and posterior nodes and had the lowest PLV of all the clusters (Figures 3C,D). Clusters 2 and 4 also reflected activity primarily in anterior and posterior nodes, with minor activity in the central or midline areas; these clusters also had higher PLV than cluster 1, but lower than cluster 3. Cluster 3 represented primarily central and midline activity and had a PLV significantly higher than the other clusters. All three filtering strategies we used also showed a consistent pattern of significant theta band activity primarily in anterior and posterior sites. This may indicate that the experimental factors of Social setting resulted in marked enhancement of between-participant theta PLV in anterior and posterior areas.

## Discussion

The present study has shown that a combination of emotional arousal and co-presence in groups mediate religious believers' perception of ritual efficacy, marked by distinct neural signatures.

Individual neural and behavioral responses linked emotional arousal and visual attention to how religious believers rated ritual efficacy. Alpha power significantly decreased along with perceptual ratings of efficacy from low, medium, to high. Alpha within-participant PLV showed a similar, likely related, trend. These neural markers for perceived efficacy were largely unaffected by social setting, indicating that participants' initial emotional responses to ritual performances were not influenced by the presence of others.

Previous research has linked alpha power suppression to individual participants' heightened cortical arousal and attentional engagement, notably when they are presented with arousing images of pain, mutilation, disgust, and fear (Aftanas et al., 2001; Wright et al., 2004; Sarlo et al., 2005; Schienle et al., 2006). Beta oscillations have similarly been associated with visual attention particularly as it is sustained over time (Gross et al., 2004; Escoffier et al., 2015). The enhanced arousal and attention, respectively marked by alpha and beta decrease in power, suggests that the dramatic spectacle of Chinese spirit-medium rituals may leverage on the emotional response they trigger in religious believers. This increases perceived efficacy and captures attentional resources.

Neural synchrony results indicated that participants in the group social setting showed a different pattern of within- and between-participant phase synchronization in beta and theta bands, compared to those who watched rituals alone. Variance in efficacy ratings of participants in a group was also greatly reduced compared to those who were tested individually. This indicated a shared neural and behavioral signature in groups, likely reflecting how co-presence in groups socially tuned cognitive processes. The remainder of this discussion covers aspects of this signature.

First, low-beta within-participant PLV was enhanced in the individual compared to group social setting. High-beta within-PLV was also enhanced for individuals compared to groups, for only medium and high efficacy rituals. Alpha and beta PLV, especially in anterior and posterior cortical regions, have been linked to top-down control of attention. In cued attention tasks alpha and beta synchrony enhancement marks the engagement of top-down processes that block distractor stimuli during the effortful attending to a target (Sharp et al., 2010; Sacchet et al., 2015). These within-participant PLV results indicate that participants engage these top-down processes more when viewing rituals alone. In groups however, they are less reliant on controlled top-down attentional processes to stay focused on the rituals. The combination of co-presence and arousal might boost the perceived relevance of the rituals by signaling their social significance, making they more likely to capture attention without effortful control (Indovina and Macaluso, 2007).

Second, our findings demonstrated a heightening of between-participant theta phase synchronization over anterior and posterior regions in group social contexts as compared to individual context for low efficacy rituals. These results were not artifacts of exposure to a shared stimuli leading to common neural responses. Individual participants were analyzed as virtual triads, which saw the same sensory input and experimental time-line as a matching triad in the group condition. This held these parameters constant across group and individual settings, and ensured that differences in social setting alone explained the effect on theta responses.

In group setting, the pattern of enhanced theta synchrony and higher agreement in efficacy perceptual ratings is likely a signature of a social-tuning effect. Studies indicate that theta synchrony indexes a range of processes for integrating sensory and social information with prior experience. These functions include cognitive control over action monitoring, pre-stimulus expectation, and memory in social settings (Mizuhara and Yamaguchi, 2007; Cavanagh et al., 2009; Lindenberger et al., 2009; Slagter et al., 2009; Min and Park, 2010; Yun et al., 2012). Furthermore, both within- and between-participant theta phase synchronization is generally prominent in social settings (Yun et al., 2012). For example, between-participant theta synchronization was observed only when participants were instructed to anticipate and imitate another's actions, in contrast to spontaneous coordination (Dumas et al., 2010). Similarly, between-brain theta coupling also preceded musicians' anticipation of both the onset and rhythm during coordinated play (Lindenberger et al., 2009). This suggests that heightened between-participant theta synchrony during group co-presence may influence perceptual attention and interpretation of events. In other words, between-participant theta synchrony may be an underlying mechanism for social-tuning effects on group perception.

Co-presence activation of cognitive processes underlying a social-tuning effect likely drove religious believers' higher perceptual ratings of efficacy in groups as compared to individuals, even for rituals rated as low arousal by naïve non-believers. While the exact interplay of social tuning processes supported by theta remains to be determined, they are likely the foundation for the emergence of the kind of shared meaning and representation that are central to the social experience of rituals.

## Theoretical implications and future directions

Our study is the first to discover neurological signatures for religious believers' perceptions of ritual efficacy, revealing a complex interaction of group and individual effects in the experience of communal rituals. These neurophysiological signatures reveal critical nuances in how collective rituals are influenced by both co-presence and arousal.

The humanities and social sciences have long speculated that “socially shared reality” in groups involves “the experience of common inner states” (Echterhoff et al., 2009). For over a century, theorists from Durkheim (1995) to Collins (2004) have asserted that communal rituals heighten emotions and somehow synchronize participants' behavior and inner states. However, as Douglas Marshall has pointed out, although there has been general agreement over the effects of collective rituals, there has been little consensus over the underlying mechanism (Marshall, 2002). Debates have raged over whether co-presence alone or shared symbolism influenced participants' perceptions. Likewise, behavioral experiments have shown that psychological proximity due to sitting side-by-side triggers perception of collective attention, which socially tunes emotions and memory of an event because of “deeper cognitive processing” (Shteynberg, 2017). These long-standing questions over how co-presence influences collective experience have been difficult to resolve without evidence for how the brain processes information in individual and group setting.

Our discovery of between-participant neural synchrony in groups is a likely underlying mechanism for social tuning and the convergence in perceptual ratings of ritual efficacy. Co-presence in group social setting also likely signals the significance of an event, attenuating the need for top-down engagement. On the other hand, spectral power markers showed that highly arousing ritual spectacle drove individual attention and perceptions of ritual efficacy, only tangentially influenced by group setting. Collective rituals thus have communal and individual dimensions, explaining how mass gatherings may foster social cohesion without necessarily requiring group-size scaling limitations of direct face-to-face interaction. This could have implications for the scaling computability of synchrony in large groups.

Further investigation of explicit face-to-face communication and interaction could shed further light on the specific cultural content of this group perspective. A limitation in the current study was that we examined co-presence alone, without having participants in the group setting directly communicate with each other. This was so that we could differentiate between stimulus-related interpersonal synchrony across individually tested participants compared to synchrony related to co-presence. Additional studies have shown that face-to-face interaction and communication not only generates synchrony between two people but also predicts mutual prosociality (Jiang et al., 2012, 2015; Hu et al., 2017). Both successful communication and memory encoding of narrative content in story segments have also been shown to produce synchrony between participants (Hasson et al., 2008; Dehghani et al., 2017). Future research on direct communication and common memory could elucidate differences between co-presence and symbolic interactionism.

## Conclusion

Together, these results indicate that the emotionality of spirit-medium rituals drives individual perceptions of the degree of efficacy, spirit-medium possession, and intentional agency. Perception of higher efficacy was marked by alpha and beta power suppression largely unaffected by group social setting. At the same time, co-presence in groups not only signaled the significance of an event, engaging bottom-up attention even when cultural cues were unclear, but also enhanced agreement in religious believers' perceptions of ritual efficacy. This social tuning of group perspective was supported by between-participant theta phase synchrony in groups. These findings are the first neurophysiological evidence of common mental states produced within groups during interpretations of cultural meaning.

## Author contributions

PC and NE: contributed equally to all aspects of the work with the following additional co-author contributions. AC: data collection. YM: data processing and statistical analysis. HW: editing. CG: planning. JJ: grant administration.

### Conflict of interest statement

The authors declare that the research was conducted in the absence of any commercial or financial relationships that could be construed as a potential conflict of interest.

## Supplementary material

Supplementary Video 1 Example of High Efficacy Ritual.

Supplementary Video 2 Example of Medium Efficacy Ritual.

Supplementary Video 3 Example of Low Efficacy Ritual.
