# Supplementary material for: Groups and Emotional Arousal Mediate Neural Synchrony and Perceived Ritual Efficacy
Source: Front Psychol. 2018 Oct 26;9:2071. doi: 10.3389/fpsyg.2018.02071 (PMC6212580; doi:10.3389/fpsyg.2018.02071)
Supplement: Supplementary file 4 [file Data_Sheet_1.pdf]

Supplemental Information

EEG Power and phase synchrony analysis: Filtering strategies of 50 Hz line noise

We analyzed the data in 3 different ways. (1) we excluded phase estimates between 45 and 55 hz from analysis, (2) we applied a notch filter (48-52 Hz band-cut edges, default EEGLAB fir filter, filter order: 424) before wavelet transform, (3) lastly we used a line removal plugin that estimates the amplitude and phase of line noise and subtracts it from the data (Cleanline EEGlab plugin, http://www.nitrc.org/projects/cleanline) before wavelet transform. The between-participant synchrony analyses were then conducted as described earlier for all 3 processing strategies.

EEG statistical analysis

Scree Plots for ROI Clusters

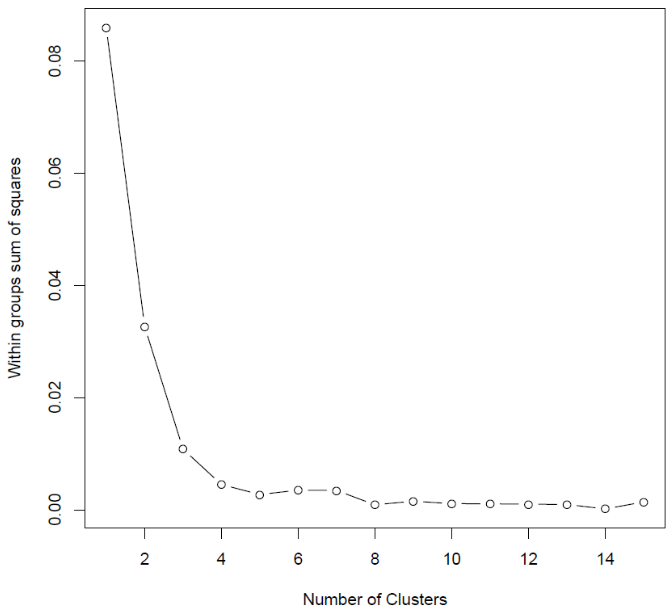

Within-Participant

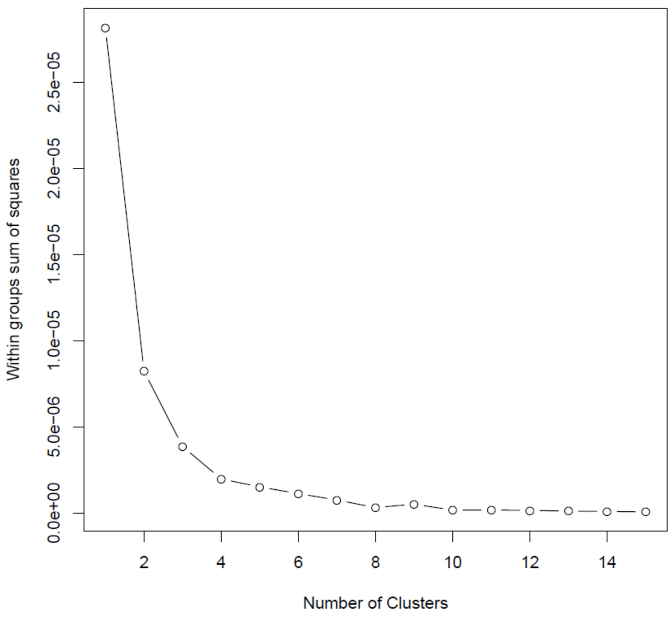

Between-Participant

Within-participant Clusters of ROI Pairs: Cluster 1 (al_ar, al_cl, al_cr, al_pl, ar_cr, ar_pl, ar_z, cl_cr, cr_cr, cr_pl, cr_pr, cr_z), Cluster 2 (al_al, al_pr, al_z, ar_pr, cl_pl, cl_pr, cl_z, pl_pr, pl_z, pr_z, z_z), Cluster 3 (ar_ar, ar_cl, pl_pl), Cluster 4 (cl_cl, pr_pr).

Between-participant Clusters of ROI Pairs: Cluster 1 (al_al, al_ar, al_pl, al_pr, ar_cr, ar_pr, pl_pl, pl_pr), Cluster 2 (al_cl, ar_ar, ar_pl, cr_pl, cr_pr, pl_z, pr_pr), Cluster 3 (cl_cl, cl_cr, cl_z, cr_cr), Cluster 4 (al_cr, al_z, ar_cl, ar_z, cl_pl, cl_pr, cr_z, pr_z, z_z).

Supplementary figures

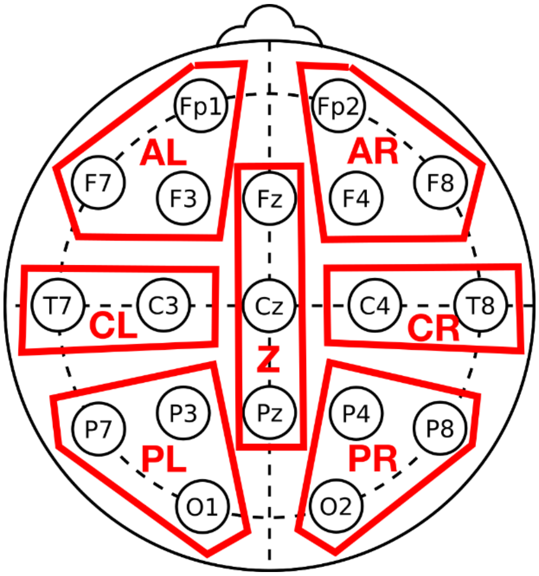

Figure S1. Topographic maps of Regions of Interest for power analysis.

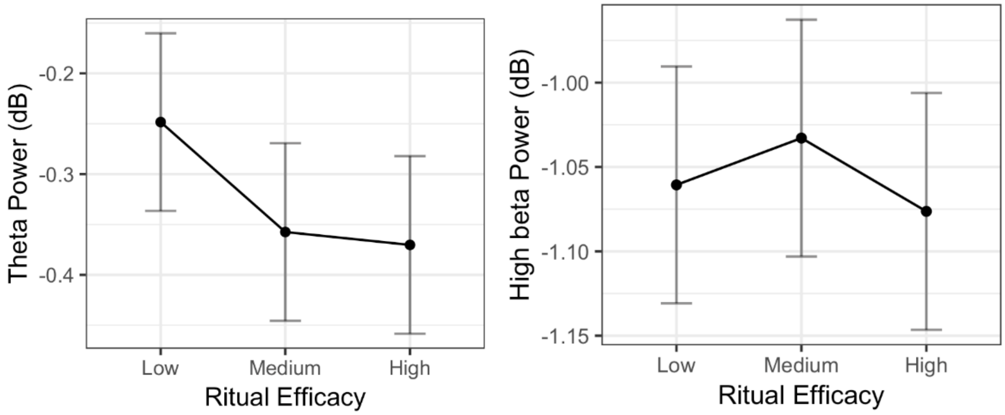

Figure S2. Theta power (left) and High beta power (right), as a function of perceive Ritual Efficacy.

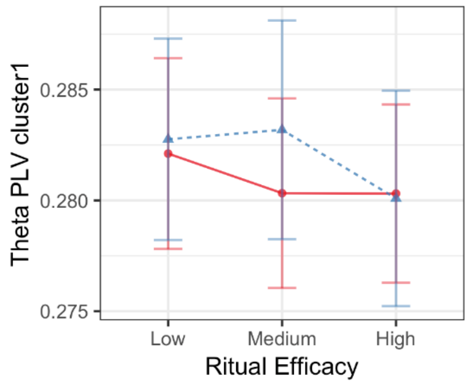

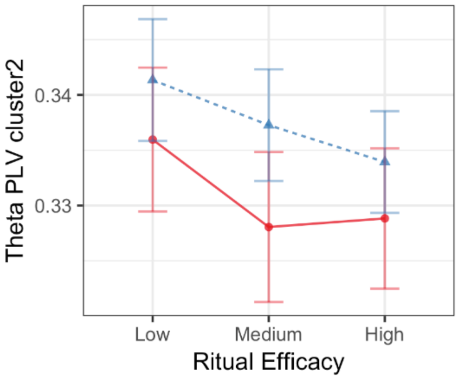

Cluster 1 *Cluster 2*

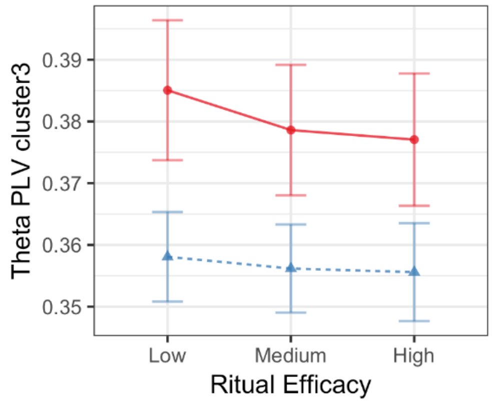

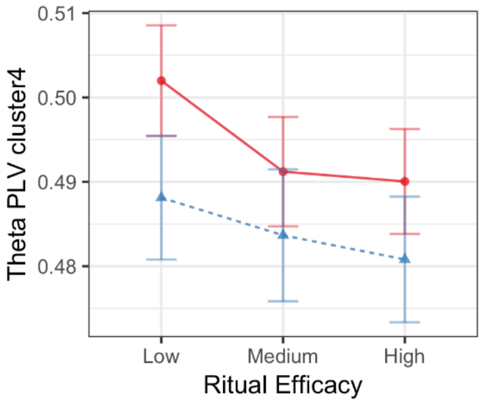

Cluster 3 *Cluster 4*

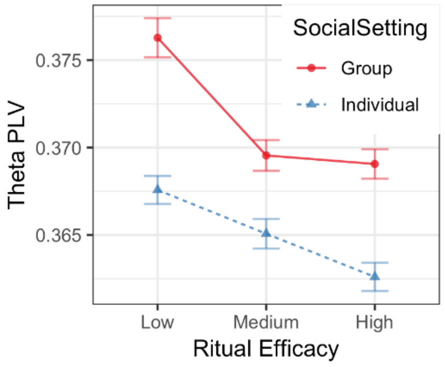

Figure S3. Within-participant Theta PLV. PLV is presented as a function of Efficacy, and Group, for each Cluster. Last plot presents the cluster average.

EEG Test Questions

[Screen 1]

In this experiment, you will be presented with a series of video clips. These clips feature Chinese spirit-mediums.

After each video clip, you will to make several ratings about the video and the medium featured. In each video the spirit-medium is the person performing the main action and the focus of the clip. There may also be several other people. Since the same medium may appear in several videos, please base your rating on your impressions of each specific clip.

We will now explain each rating. Please ask the experimenter if you need clarifications.

Press SPACE bar to continue

在这项实验中，您会看到一系列关于中国乩童的视频剪辑。
每个视频后，您将会被要求从若干方面评价视频。
所有的评价请尽量利用评定量表的全部范围，如果您认为合适，不必顾虑给出中性或极端评价。

每个视频中会出现一些人，乩童是其中的核心，担任最重要的活动。由于同一个乩童可能出现在若干个视频里，请您对每一个视频中乩童的具体表演做评价。

现在我们开始解释每一个评价。如果您需要更清楚的说明，请询问实验者。
请按 SPACE 继续。

[Screen 2: Self-Assessment Manikan scale, arousal]

In this rating scale, please select a number between 1 and 10 that best reflects how you felt while watching each video.

Select a lower number if you felt more relaxed, calm, sluggish, dull, sleepy, or unaroused emotionally.
Select a higher number if you felt stimulated, excited, frenzied, jittery, wide awake, or aroused emotionally.
Each drawing of a person represents how you felt while watching the videos.

Please press any of the number keys to move on to the next rating

What was your reaction while watching this video clip?

1 2 3 4 5 6 7 8 9 10

这个评定量表中，请在 1-10 中选择一个能最好描述您观看视频时感受的数字。

如果您觉得放松、平静、无聊、乏味、不被吸引，请选择较小的数字。
如果您觉得刺激、兴奋、狂热、狂暴、警醒，请选择较高的数字。
每个人物画像代表您观看视频时的感受。

您观看这个视频时，是怎样的反应？

1 2 3 4 5 6 7 8 9 10

[Screen 3: Self-Assessment Manikin scale, valence]
In this rating scale, please select a number between 1 and 10 that best reflects how you felt while watching each video.

Select a lower number if you felt unhappy, annoyed, unsatisfied, melancholic, despairing, or bored.
Select a higher number if you felt more happy, pleased, satisfied, contented, hopeful or relaxed.
Each drawing of a person represents how you felt while watching the videos.

What was your reaction while watching this video clip?

1 2 3 4 5 6 7 8 9 10

这个评定量表中，请在 1-10 中选择一个能最好描述您观看视频时感受的数字。

如果您觉得不开心、厌烦、不满足、忧郁、绝望、无聊，请选择较低的数字。
如果您感到高兴、愉悦、满足、开心、充满希望或放松的，请选择较高的数字。
每个人物画像代表您观看视频时的感受。

您观看这个视频时，是怎样的反应？

1 2 3 4 5 6 7 8 9 10

[Screen 4: Efficacy Rating]

In this rating scale, please select a number between 1 and 10 to rate how efficacious the ritual is in this video clip.

How efficacious is the ritual in this clip?

Not efficacious at all   Very efficacious

1 2 3 4 5 6 7 8 9 10

这个评定量表中，请在 1-10 中选择一个数字评价视频中的仪式有多灵。

如果您觉得仪式不灵，选择较低的数字。

如果您觉得仪式灵，请选择较高的数字。

您如何评价视频中的仪式有多灵？

不灵   非常灵

1 2 3 4 5 6 7 8 9 10

[Screen 5: Degree of Possession]

In this rating scale, please select a number between 1 and 10 to rate how much the medium is possessed.

Select a low number if you think the spirit-medium is not possessed at all in this clip.
Select a high number if you think the spirit-medium is very possessed in this clip.

In this segment, how much is the medium possessed?

Not possessed at all   Very possessed

1 2 3 4 5 6 7 8 9 10

这个评定量表中，请在 1-10 中选择一个数字评价乩童在多大程度上被附身。

如果您觉得视频中的乩童没有被附身，请选择较小的数字。

如果您觉得视频中的乩童被附身，请选择较大的数字。

您如何评价视频中的乩童在多大程度上被附身？

没有被附身 完全被附身

1 2 3 4 5 6 7 8 9 10

[Screen 6: Spirit-Medium’s Intentional Agency]

In this rating scale, please select a number between 1 and 10 to rate how much the medium intends to do what he is doing, how much in control of his actions he is.

Select a low number if you think the spirit-medium is does not intend to do what he is doing in this clip.
Select a high number if you think the spirit-medium is does intend to do what he is doing in this clip.

In this segment, how much does the medium intends to do what he is doing?

Does not intend at all Fully intend

1 2 3 4 5 6 7 8 9 10

这个评定量表中，请在 1-10 中选择一个数字评价乩童是否有意这么做的。

如果您觉得视频中的乩童不是有意这么做的，请选择较小的数字。

如果您觉得视频中的乩童是有意这么做的，请选择较大的数字。

你觉得乩童是有意这么做的吗？

不是有意这么做的 是有意这么做的

1 2 3 4 5 6 7 8 9 10

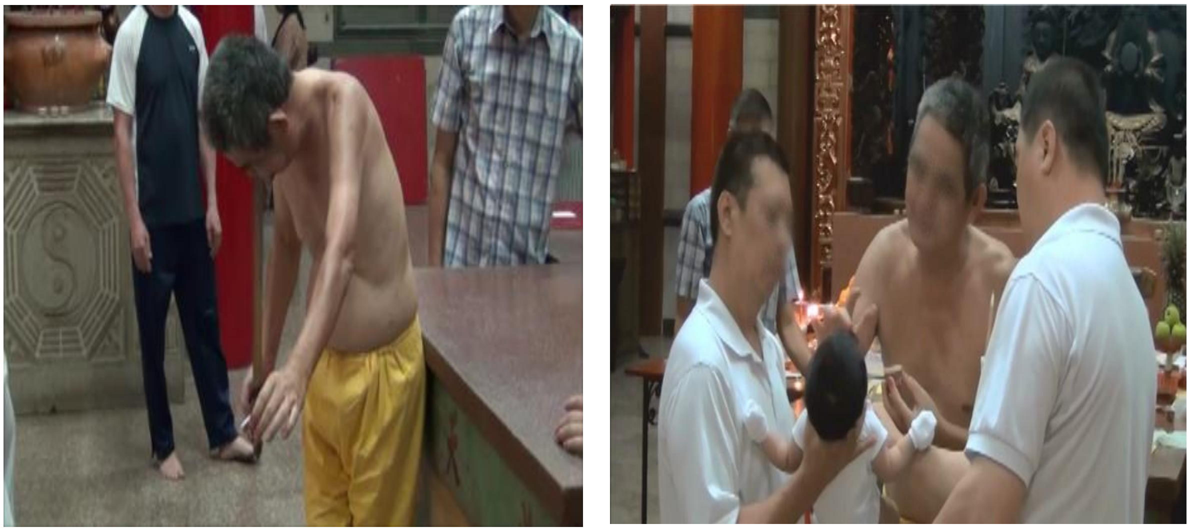

Spirit-medium cuts his tongue to collect blood to write talismans and bless a newborn
